# Exercise attenuates neurological deficits by stimulating a critical HSP70/NF-κB/IL-6/synapsin I axis in traumatic brain injury rats

**DOI:** 10.1186/s12974-017-0867-9

**Published:** 2017-04-24

**Authors:** Chung-Ching Chio, Hung-Jung Lin, Yu-Feng Tian, Yu-Chieh Chen, Mao-Tsun Lin, Cheng-Hsien Lin, Ching-Ping Chang, Chien-Chin Hsu

**Affiliations:** 1 Department of Surgery, Chi Mei Medical Center, Tainan, 710 Taiwan; 2 Department of Emergency Medicine, Chi Mei Medical Center, Tainan, 710 Taiwan; 3 Department of Biotechnology, Southern Taiwan University of Science and Technology, Tainan, 710 Taiwan; 4 Division of General Surgery, Department of Surgery, Chi Mei Medical Center, Tainan, 710 Taiwan; 5 Department of Health and Nutrition, Chia Nan University of Pharmacy and Science, Tainan, 717 Taiwan; 6 Department of Medical Research, Chi Mei Medical Center, Tainan, 710 Taiwan; 7 Meridigen Biotech Co., Ltd, Taipei, 11493 Taiwan; 8 The Ph.D. Program for Neural Regenerative Medicine, Taipei Medical University, Taipei, 110 Taiwan

**Keywords:** Brain injury, Neuroprotection, Neuroinflammation, Exercise, IL-6, HSP70, Synapsin I

## Abstract

**Background:**

Despite previous evidence for a potent inflammatory response after a traumatic brain injury (TBI), it is unknown whether exercise preconditioning (EP) improves outcomes after a TBI by modulating inflammatory responses.

**Methods:**

We performed quantitative real-time PCR (qPCR) to quantify the genes encoding 84 cytokines and chemokines in the peripheral blood and used ELISA to determine both the cerebral and blood levels of interleukin-6 (IL-6). We also performed the chromatin immunoprecipitation (ChIP) assay to evaluate the extent of nuclear factor kappa-B (NF-κB) binding to the DNA elements in the IL-6 promoter regions. Also, we adopted the Western blotting assay to measure the cerebral levels of heat shock protein (HSP) 70, synapsin I, and β-actin. Finally, we performed both histoimmunological and behavioral assessment to measure brain injury and neurological deficits, respectively.

**Results:**

We first demonstrated that TBI upregulated nine pro-inflammatory and/or neurodegenerative messenger RNAs (mRNAs) in the peripheral blood such as CXCL10, IL-18, IL-16, Cd-70, Mif, Ppbp, Ltd, Tnfrsf 11b, and Faslg. In addition to causing neurological injuries, TBI also upregulated the following 14 anti-inflammatory and/or neuroregenerative mRNAs in the peripheral blood such as Ccl19, Ccl3, Cxcl19, IL-10, IL-22, IL-6, Bmp6, Ccl22, IL-7, Bmp7, Ccl2, Ccl17, IL-1rn, and Gpi. Second, we observed that EP inhibited both neurological injuries and six pro-inflammatory and/or neurodegenerative genes (Cxcl10, IL-18, IL-16, Cd70, Mif, and Faslg) but potentiated four anti-inflammatory and/or neuroregenerative genes (Bmp6, IL-10, IL-22, and IL-6). Prior depletion of cerebral HSP70 with gene silence significantly reversed the beneficial effects of EP in reducing neurological injuries and altered gene profiles after a TBI. A positive Pearson correlation exists between IL-6 and HSP70 in the peripheral blood or in the cerebral levels. In addition, gene silence of cerebral HSP70 significantly reduced the overexpression of NF-κB, IL-6, and synapsin I in the ipsilateral brain regions after an EP in rats.

**Conclusions:**

TBI causes neurological deficits associated with stimulating several pro-inflammatory gene profiles but inhibiting several anti-inflammatory gene profiles of cytokines and chemokines. Exercise protects against neurological injuries via stimulating an anti-inflammatory HSP70/NF-κB/IL-6/synapsin I axis in the injured brains.

## Background

Mounting evidence indicates that inflammation is a major contributor to secondary brain injury caused by a traumatic brain injury (TBI) [1]. Neuroinflammatory events, including glia activation, leukocyte recruitment, and mediator overexpression actively participates in the pathogenesis of TBI [2–4]. Despite previous evidence for the favorable effects of exercise preconditioning (EP) in neurorehabilitation after a TBI, it remains unknown the detailed mechanisms exerted by EP or post-TBI inflammation have been limited [5–7].

Serum levels of heat shock protein 70 (HSP70) measured after an initial TBI correlate with survival [8]. HSP70 expression is upregulated both in human and in rodent TBI, which suggests that HSP70 upregulation is an important endogenous neuroprotective response after a TBI [9]. After a TBI, interleukin-6 (IL-6) is also elevated in serum [10], cerebrospinal fluid [11], and brain tissue [12] and is considered a biomarker of TBI outcome [13–15]. In a rodent TBI model, depletion of IL-6 exacerbates outcomes of a TBI, whereas overexpression of IL-6 shows a more rapid healing and recovery after a TBI [16]. Nuclear factor kappa-B (NF-κB) participates in inflammation, immune responses, and cell survival by affecting many different genes including IL-6 [17]. Production of several nerve growth factors including synapsin I can be stimulated by IL-6 [18]. According to the Ingenuity Pathway Analysis (IPA) software program [19], an HSP70/NF-κB/IL-6/synapsin I axis exists during the inflammatory response. However, additional studies are needed to ascertain whether the axis exists and plays a critical inflammatory signaling system in the injured brains. In fact, EP protects against ischemic stroke [20], heat stroke [21], and spinal cord injury [22] in rodents by inducing overexpression of HSP70. Again, it remains unknown whether EP attenuates neurological injury after a TBI by affecting on the proposed HSP70/NF-κB/IL-6/synapsin I signaling system.

In the present study, in addressing the questions mentioned above, we first used quantitative real-time PCR (qPCR) assay to determine which cytokines and chemokines in the blood are significantly altered after a TBI in rats with or without EP. The highly expressed molecules detected in the injured brain tissue are detectable in the peripheral blood [23, 24]. Second, we aimed to delineate whether a Pearson correlation coefficient exists between the blood levels of certain highly detectable genes (e.g., IL-6 by qPCR) and brain levels of HSP70 (measurement by Western blotting analysis). The chromatin immunoprecipitation (ChIP) assay was also used to evaluate the expression of NF-κB binding to the DNA element in the IL-6 promoter regions in ipsilateral cortex of rats with or without EP. Finally, we used Western blotting method to assess the synapsin I expression in the ipsilateral cortical regions of rats with or without EP. There are twofold of the present study. The one is to elucidate whether HSP70/NF-κB/IL-6/synapsin I axis is a critical inflammatory signaling system in the damaged brain following a TBI. Second, we try to understand whether EP attenuates neurological injury in a rat model by acting via this axis.

## Methods

### Study design

Eight groups of rats were randomly assigned as the following: (i) no exercise-preconditioned sham controls received siRNA-vector (EP^−^ + sham + siRNA-vector); (ii) no exercise-preconditioned sham controls received siRNA-HSP70 (EP^−^ + sham + siRNA-HSP70); (iii) exercise-preconditioned sham controls received siRNA-vector (EP^+^ + sham + siRNA-vector); (iv) exercise-preconditioned sham controls received siRNA-HSP70 (EP^+^+ sham + siRNA-HSP70); (v) no exercise-preconditioned TBI rats received siRNA-vector (EP^−^ + TBI + siRNA-vector); (vi) no exercise-preconditioned TBI rats received siRNA-HSP70 (EP^−^ + TBI + siRNA-HSP70); (vii) exercise-preconditioned TBI rats received siRNA-vector (EP^+^ + TBI + siRNA-vector); and (viii) exercise-preconditioned TBI rats received siRNA-HSP70 (EP^+^ + TBI + siRNA-HSP70) (Table 1).

**Table 1:** Experimental groups included in the study

Experimental groups	Biochemical, histological, and functional evaluation
Sham operation 1.EP^−^ + sham + siRNA-vector 2.EP^−^ + sham + siRNA-HSP72 3.EP^+^ + sham + siRNA-vector 4.EP^+^ + sham + siRNA-HSP72	
	1.Western blotting: HSP70 and synapsin I
	2.Neurological motor function assay
	3.Cerebral contusion assay
	4.BBB permeability and brain edema assay
Traumatic brain injury (TBI) 1.EP^−^ + TBI + siRNA-vector 2.EP^−^ + TBI + siRNA-HSP72 3.EP^+^ + TBI + siRNA-vector 4.EP^+^ + TBI + siRNA-HSP72	5.Immunofluorescence triple staining to identify neuron apoptosis
	6.qPCR cytokines and chemokines array
	7.NF-κB ChIP assay
	8.IL-6 ELISA

*EP* ^*+*^ exercise preconditioning, *EP* ^−^, non-exercise preconditioning, *siRNA-vector* pSUPER interfering RNA delivery media, *siRNA-HSP70*, pSUPER small interfering RNA expressing HSP70

In experiment 1, the optical density (O.D.) values of HSP70 of ipsilateral hemisphere were determined 3 days after a TBI or a sham operation in all groups.

In experiment 2, 1 day before, 1–3 days after a TBI or a sham operation, we determined the neurological motor functions for all eight groups of rats.

In experiment 3, in all eight groups of rats 3 days after the operation, we measured both cerebral contusion and Evans Blue extravasations.

In experiment 4, both neuronal loss and apoptosis were determined by using immunofluorescence stain in all eight groups 3 days after a TBI.

In experiment 5, qPCR arrays were used to evaluate blood levels of inflammatory cytokines and chemokines in six groups as follows: (i) EP^−^ + sham + siRNA-vector group, (ii) EP^+^ + sham + siRNA-vector group, (iii) EP^+^ + sham + siRNA-HSP70 group, (iv) EP^−^ + TBI + siRNA-vector group, (v) EP^+^ + TBI + siRNA-vector group, and (vi) EP^+^ + TBI + siRNA-HSP70 group.

In experiment 6, the correlation between IL-6 and HSP70 in the peripheral blood and brain tissues (evaluated by qPCR or ELISA and Western blot), NF-κB transcription activation (evaluated by ChIP), and synapsin I (evaluated by Western blot) in six groups as follows: (i) EP^−^ + sham + siRNA-vector group, (ii) EP^+^ + sham + siRNA-vector group, (iii) EP^+^ + sham + siRNA-HSP70 group, (iv) EP^−^ + TBI + siRNA-vector group, (v) EP^+^ + TBI + siRNA-vector group, and (vi) EP^+^ + TBI + siRNA-HSP70 group.

### Animals

We purchased male Wistar rats (300–320 g) from BioLASCO Taiwan Co., Ltd. (Taipei, Taiwan). The BioLASCO’s policies on the care and use of laboratory animals were followed. The Institutional Animal Care and Use Committee of Chi Mei Medical Center approved the experiments (IACUC Approval No. 101122405). All efforts were made to minimize animal suffering and reduce the number of rats used. The rats were housed under controlled laboratory conditions with a 12-h light/dark cycle, a temperature of 22 ± 1 °C, and a humidity of 60–70% for at least 1 week before surgery or drug treatment. We provide enough chow and unlimited fresh drinking water for rats.

### Exercise training protocol

The exercise training protocol was implemented according to the procedure described previously [22]. Animals were trained on a treadmill 5 days a week for 3 weeks.

### Induction of TBI

TBI was induced using a fluid percussion injury device on rats placed in a Kopf stereotaxic frame (Kopf Instruments, Tujunga, CA, USA) detailed previously [25]. Each injured or sham-injured rat was housed individually and closely evaluated for behavioral recovery immediately after a TBI.

### Intracortical injection of recombinant pSUPER plasmid expressing HSP70 siRNA construction

pSUPER vector (Oligoengine, Seattle, WA, USA) contains H1 RNA polymerase III promoter, which can direct the synthesis of the siRNA-like transcript. The target sequence for HSP70 (Gen Bank Accession No. NM_031971) was chemically synthesized (Tri-I Biotech, Taipei, Taiwan) as complementary oligonucleotides [26]. A BLAST search of the Rattus genome database was done to ensure that the sequence did not target another gene transcript (http://blast.ncbi.nlm.nih.gov/Blast.cgi). The clone HSP70 target sequence was sequence confirmed using a DNA sequencer as shown in Chang et al. [22]. Three days before a TBI or a sham operation, rats were anesthetized using sodium pentobarbital (25 mg/kg, i.p.) (Sigma-Aldrich, St. Louis, MO, USA) and placed in a Kopf stereotaxic frame. An acute dose of pSUPER plasmid expression HSP70 siRNA (siRNA-HSP70) (12.5 μg/rat) in 25 μl of pSUPER RNAi delivery media (siRNA-vector) was microinjected into the frontal cortex at 0.5 μl/min flow rate using a microinfusion pump (CMA 100, Carnegie Medicine AB, Stockholm, Sweden) according to the coordinates of the atlas of Paxinos and Watson [27]: from bregma, anteroposterior (AP) −3 mm, lateral (L) 4 mm, and dorsoventral (DV) 2 mm. After the injection was completed, the cannula was left in place for 5 min and then removed at 1 mm/min.

### Neurological and motor function evaluation

The acute neurological injury was assessed in all rats the day before and 1–3 days after surgery using a modified neurological severity score, a composite of the motor, sensory, and reflex test scores [28]. The higher of the score was given, the more severe of the injury. The degrees of the inclined plane in which the rats can hold were defined as the extents of limb muscle strength [29]. The angle of the inclined plane was increased or decreased in 5^o^ increments to determine the maximal angle in which a rat could hold to the plane. The data for each were the mean of left- and right-side maximal angle. Neurological and motor functions were examined and scored blindly.

### Cerebral contusion assay

The triphenyl tetrazolium chloride (TTC) (Sigma-Aldrich, St. Louis, MO, USA) staining procedures are used for determining cerebral ischemia extent caused by a TBI [30]. An image analysis system (Image Pro Plus 4.50.29, Media Cybernetics, Inc., Silver Spring, MD, USA) measured the volume of the contusion as revealed by negative TTC stains. The volume of infarction was calculated as 1 mm (thickness of the slice) × [sum of the infarction area in all brain slices (mm^2^)].

### Evans Blue extravasation and brain water contents

Evans Blue dye (Sigma-Aldrich, St. Louis, MO, USA) was administered intravenously at 3 days after the onset of a TBI. After the dye had circulated 2 h, under deep anesthesia, the rats were transcardially perfused with physiological saline containing 10 U/ml of heparin. Brains were removed for determination of Evans Blue extravasation or brain water contents according to the methods described previously [31].

### Protein analysis and quantification

At predetermined time points after a TBI, the rats were anesthetized with sodium pentobarbital and perfused via cardiac puncture with 0.1 M of phosphate buffered saline (PBS; pH 7.4). Western blotting of the brain tissues samples was done 3 days after a TBI. The methods used for determination of brain expression of HSP70 (1:1000; Enzo Life Sciences, Farmingdale, NY, USA), synapsin I (1:4000; GeneTex, Inc., San Antonio, TX, USA), and β-actin (1:4000; Santa Cruz Biotechnology, Santa Cruz, CA, USA) were modified from our previous study [22].

### Immunofluorescence staining

Rats were perfused with paraformaldehyde (Sigma-Aldrich, St. Louis, MO, USA) solution in PBS (pH 7.4) at 3 days following a TBI, and brains were removed and fixed with 10% buffered formalin (Sigma-Aldrich, St. Louis, MO, USA). Ten-micrometer sections were cut from paraffin-embedded tissue blocks. The sections were xylene- and ethanol-treated for deparaffinization and dehydration. Triple immunofluorescence was performed using the neuronal marker anti-NeuN (1:100, Abcam, Cambridge, UK) and 4′,6-diamidino-2-phenylindole (DAPI; Sigma-Aldrich, St. Louis, MO, USA) in combination with terminal deoxynucleotidyl transferase-mediated dUTP nick-end labeling (TUNEL) (Roche Inc., Mannheim, Germany), as previously described [30, 32]. The sections were coverslipped with the mounting medium (DAKO’s fluorescent mounting medium; Agilent Technologies, Denmark). For negative control sections, all the procedures were without the primary antibody. Images were made with a Carl Zeiss upright fluorescence microscope (Carl Zeiss, Jena, Germany) using Plan-Apochromat 63×/1.4 oil and Plan-Neofluar 40×/1.30 oil objectives and analyzed with an Axiovision image analysis software (Carl Zeiss, Jena, Germany). The NeuN/DAPI/TUNEL triple-labeled cells were calculated in five coronal sections from each rat and counted in at least six rats per group and expressed as the mean number of cells per section.

### Blood sampling, RNA purification, and gene analysis

For messenger RNA (mRNA) analysis, we killed the animals with an overdose of sodium pentobarbital and collected the blood samples via cardiac puncture. Blood was collected with heparinized RNA protect animal blood tubes (Qiagen, Valencia, CA, USA) to prevent the blood from coagulating. Whole blood was separated into supernatant and pellet fractions within 30 min after blood was collected. Then pellet fractions were discarded, and supernatant were stored at −80 °C. RNA was isolated using Trizol reagent (Invitrogen, Grand Island, NY, USA) and manufacturer’s recommended protocol. Total blood RNA was subsequently treated with DNase I (Qiagen) to remove any traces of contaminating DNA and further purified using an RNeasy Protect Animal Blood Kit (Qiagen). Total RNA concentration was determined by a spectrophotometric optical density measurement (260 nm [OD260] and 280 nm [OD280]). For each sample tested, the ratio between the spectrophotometric readings at 260 and 280 nm (OD260/OD280) in all samples ranged between 1.8 and 2.2. One microgram (1 ug) of high-quality total RNA (RNA integrity number (RIN) >7) was then reverse transcribed using the First Strand Synthesis kit (Qiagen) and reverse transcriptase with reverse transcription cocktail (Molecular Genetic Resources, Tampa, FL, USA) to obtain cDNA and subsequently loaded onto a RT^2^ Profiler^TM^ PCR Array Rat Cytokines & Chemokines profiler array according to manufacturer’s instructions (Qiagen). Quantitative real-time polymerase chain reaction (qPCR; with gene-specific primers and SYBR green assay) was performed to quantify the genes encoding 84 cytokines and chemokines. QPCR was performed in triplicate in six separate experiments on an Applied Biosystems 7500 system (Applied Biosystems, Foster City, CA, USA). Qiagen’s online web analysis tool (http://www.qiagen.com/us/shop/genes-and-pathways/data-analysis-center-overview-page/) was utilized to produce comparative cluster gram, and fold change was calculated by determining the ratio of mRNA levels to control values using the ΔCt method (2^−ΔΔCt^). The RT^2^ Profiler™ PCR Array Rat Cytokines & Chemokines profiles the expression of five sets of commonly used housekeeping genes. The five sets of housekeeping genes used in our assessing include B2m (beta-2 microglobulin, a cytoskeletal protein), Hprt1 (hypoxanthine phosphoribosyltransferase 1, involved in the metabolic salvage of purines), Rplp1 (ribosomal protein large P1, involved in structural constituent of ribosome), Ldha (lactate dehydrogenase A, involved in catalytic activity), and Actb (actin beta, a cytoskeletal structure protein). This commercialized array kit can be easily used to identify genes with a constant level of expression among our different experimental conditions for use in normalizing our 84 relative gene expression profiling experiment. All data from EP^−^ + sham + siRNA-vector group was normalized to 100%. The PCR conditions used hold for 10 min at 95 °C, followed by 40 cycles of 15 s at 95 °C, 40 s at 55 °C, and 30 s at 72 °C.

### Ingenuity pathway analysis

Lists of differentially expressed genes (DEG) from qPCR analysis were generated using a cutoff >2 fold between EP^−^ + TBI + siRNA-vector vs. EP^+^ + TBI + siRNA-vector groups and used as the input for QIAGEN’s Ingenuity® Pathway Analysis (IPA®, Redwood City, CA, USA; https://www.qiagenbioinformatics.com/products/ingenuity-pathway-analysis/), and the top five canonical pathways were observed. Canonical pathways that were enriched in the DEG datasets were determined. From the IPA library, we identified the canonical molecular pathways that were most significant to the dataset. We further analyzed the genes from the dataset that were related to canonical pathways.

### Enzyme-linked immunosorbent assay

IL-6 levels of animals were measured by enzyme-linked immunosorbent assay (ELISA) (BD Biosciences, San Jose, CA, USA) at 3 days after a TBI, an exercise preconditioning or an HSP70 siRNA injection. The frozen lesioned side of rat’s cortex was mechanically homogenized and centrifuged at 12,000 rpm for 10 min at 4 °C. The cerebral levels of inflammatory cytokines were quantified using specific ELISA kits for rats according to the manufacturers’ instructions. The inflammatory mediators were expressed as picogram per milligram protein (pg/mg protein).

### Chromatin immunoprecipitation assay

Chromatin immunoprecipitation (ChIP) assay (EpiTect ChIP qPCR Assay kit; Qiagen) was performed to evaluate the extent of NF-κB binding to the DNA elements in the IL-6 promoter regions respectively using “EpiTect ChIP qPCR Assays kit” from Qiagen. ChIP assays were done as previously described [33]. Ipsilateral lesion side cortical tissue was exposed to 1% formaldehyde to cross-link with DNA proteins. We added glycine to each sample tube to quench unreacted formaldehyde. Cortical tissue was broken open with a sodium dodecyl sulfate (SDS) lysis buffer containing Protease Inhibitor Cocktail II (Calbiochem: Merck Millipore, Darmstadt, Germany). We sheared the chromatin to a manageable size by using a sonicator. Generally, between 200 and 1000 bp of DNA is used to achieve a high degree of resolution during the detection step. We added Protein G Agarose (Millipore, Billerica, MA) to each IP tube to remove proteins or DNA. We used an anti-NF-κB Ab (Millipore) or rabbit IgG (Millipore) to immunoprecipitate the precleared chromatin. It was then incubated overnight at 4 °C while being rotated. Protein G Agarose was added to each IP tube and incubated for 1 h at 4 °C with rotation to collect the antibody/antigen/DNA complex. After four consecutive washes using four different wash buffers, the DNA from the DNA-protein complexes from all the samples, including the input and negative control, was reverse cross-linked by incubation with 2 μL of Proteinase K for 2 h at 65 °C. DNA was purified to remove chromatin proteins and to prepare it for the detection step. Primers specific for IL-6 promoters were used to determine the extents of both immunoprecipitated DNA and quantitative PCR. The following IL-6 primers were used: forward, 5′-GCG ATG GAG TCA GAG GAA AC-3′, and reverse 5′-TGA GGC TAG CGC TAA GAA GC-3′. Results are presented as a percentage of input.

### Statistical analysis

Data are mean ± standard deviation (SD) and were analyzed using one-way analysis of variance (ANOVA) with Fisher’s post hoc test where appropriate. Analyses for behavioral variables used Student’s unpaired *t* test to compare variables between groups. Bonferroni’s multiple comparison test was then used when appropriate to determine post hoc significance at individual time points. Sigma Plot version 12.0 software (Systat Software Inc., San Jose, CA, USA) was used to analyze all data. A *p* value of less than 0.05 was considered to be significant.

## Results

### Brain levels of HSP70 were attenuated in EP^+^ rats treated with small interfering RNA

**Fig. 1:**
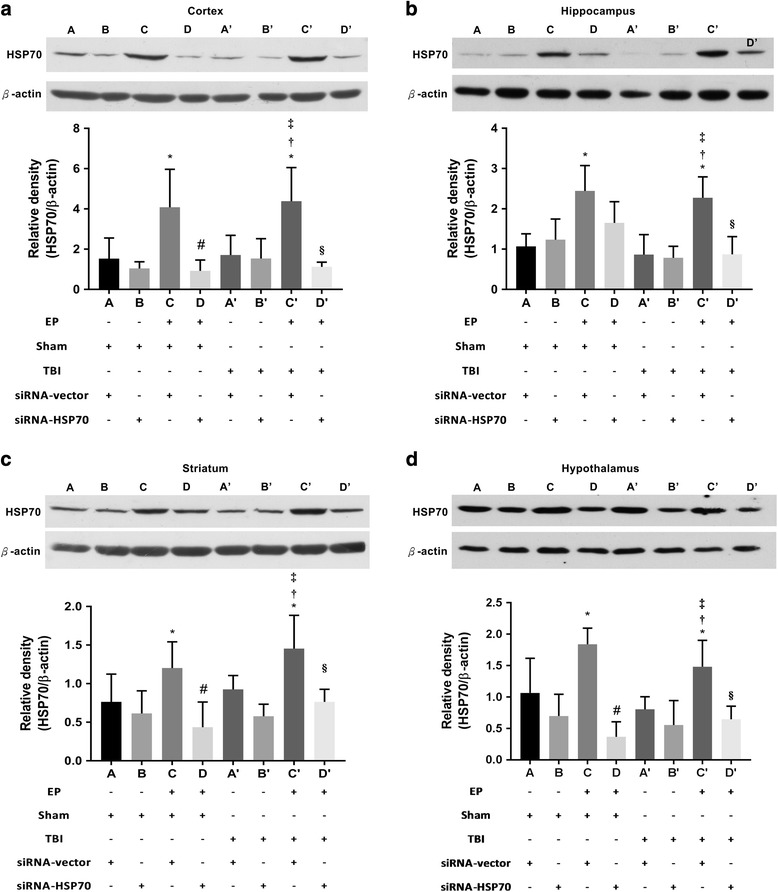
HSP70 expression was higher in the ipsilateral frontal cortex (**a**), hippocampus (**b**), striatum (**c**), and hypothalamus (**d**) in exercise-preconditioned (EP^+^) rats with traumatic brain injury (TBI) or without TBI (sham). *A*: EP^−^ rats were given a sham operation and intracerebral administration of siRNA-vector (EP^−^ + sham + siRNA-vector). *B*: EP^−^ rats were given a sham operation and intracerebral siRNA-HSP70 (EP^−^ + sham + siRNA-HSP70). *C*: EP^+^ + sham rats were given siRNA-vector (EP^+^ + Sham + siRNA-vector). *D*: EP^+^ + sham rats were given intracerebral siRNA-HSP70 (EP^+^ + sham + siRNA-HSP70). *A’*: EP^−^ + TBI^+^ rats were given intracerebral siRNA-vector (EP^−^ + TBI + siRNA-vector). *B’*: EP^−^ + TBI^+^ rats were given intracerebral siRNA-HSP70 (EP^−^ + TBI + siRNA-HSP70). *C’*: EP^+^ + TBI^+^ rats were given intracerebral siRNA-vector (EP^+^ + TBI + siRNA-vector). *D’*: EP^+^ + TBI^+^ rats were given intracerebral siRNA-HSP70 (EP^+^ + TBI + siRNA-HSP70). Cerebral expression of HSP70 was assessed by Western blotting 3 days after a TBI or a sham operation. The gels presented are representatives of results from three separate experiments. Densitometry readings of gel bands expressed as arbitrary units of relative intensities to that of EP^−^ + TBI^−^ + siRNA-vector control. Values represent mean ± SD of three separate experiments. *P < 0.05 for *C* or *C'* vs. *A*; #*P* < 0.05 for *D* vs. *C*; †*P* < 0.05 for *C’* vs. *A’*; ‡*P* < 0.05 for *C’* vs. *B’*; and §*P* < 0.05 for *D’* vs. *C’*

As shown in previous studies [20–22], HSP70 expression was significantly higher in ipsilateral brain regions including the frontal cortex (Fig. 1a), hippocampus (Fig. 1b), striatum (Fig. 1c), and hypothalamus (Fig. 1d) in both the (EP^+^ + TBI + siRNA-vector) and the (EP^+^ + sham + siRNA-vector) rats evaluated 3 days post-injury. In addition, the increased cerebral HSP70 expression caused by EP^+^ were significantly attenuated by an acute dose of pSUPER plasmid expression of HSP70 small interfering RNA (siRNA-HSP70) (5 μg/rat in 5 μl of pSUPER RNAi delivery media [siRNA-vector]) into the injured cortex to induce gene silencing [21, 26, 34]. Western blotting analyses revealed that such an intracerebral injection of siRNA-HSP70, but not siRNA-vector, significantly reduced HSP70 expression in the ipsilateral brain tissue of EP^+^ + TBI + siRNA-HSP70 and EP^+^ + sham + siRNA-HSP70 rats (Fig. 1).

### The outcomes of EP^+^ + TBI rats were better

**Fig. 2:**
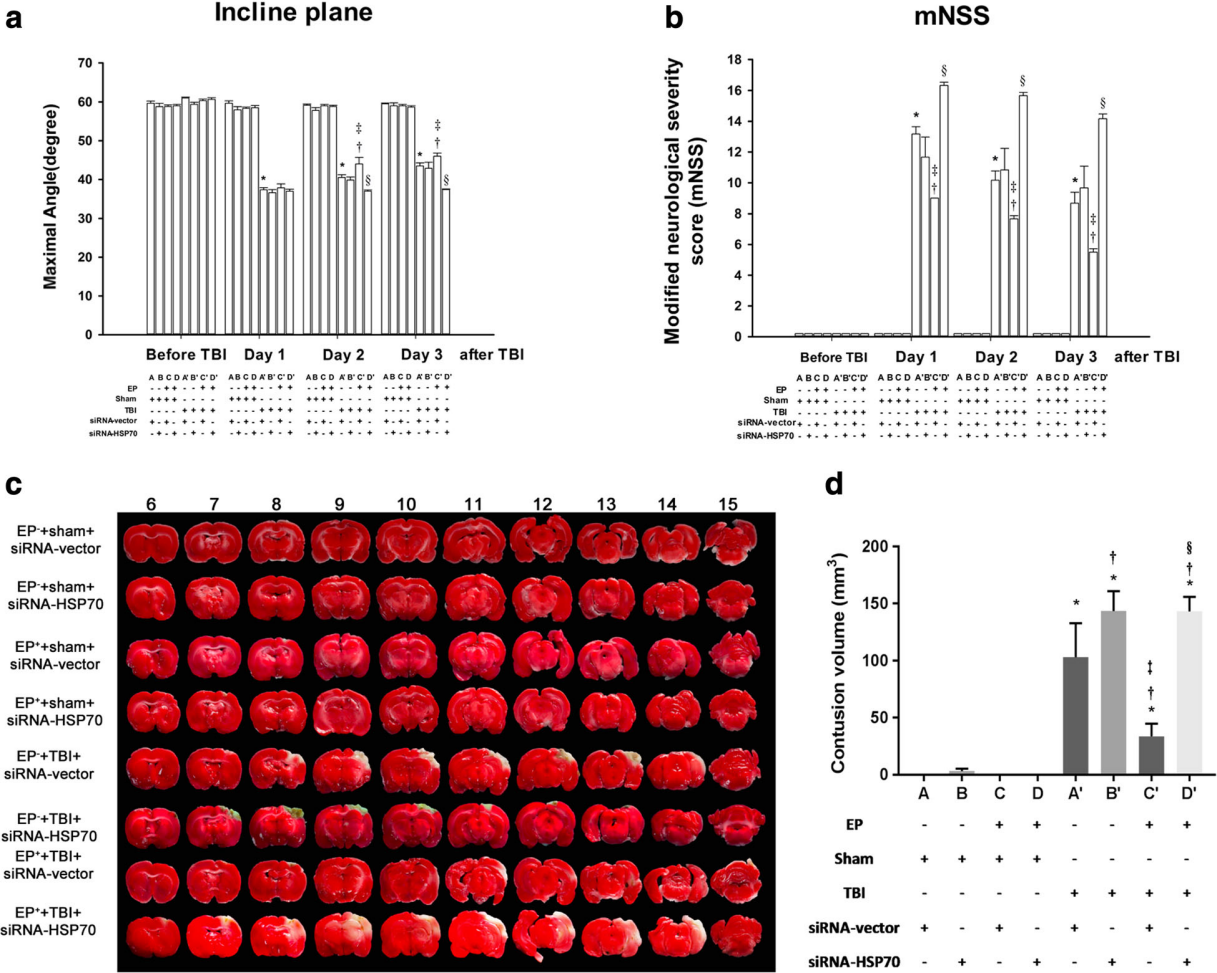
TBI-induced neurological motor deficits, brain contusion. Both the inclined plane test (**a**) and the modified neurological severity score (mNSS) (**b**) were used to assess the neurological function. The tetrazolium chloride (TTC) stains are representatives of brain contusion results (**c**). Data are presented as mean ± SD (*n* = 8 per group) (**d**). *P < 0.05 for *A’* or *B’* or *C’* or *D’* vs. *A*; †*P* < 0.05 for *B’* or *C’* or *D’* vs. *A’*; ‡*P* < 0.05 for *C’* vs. *B’*; and §*P* < 0.05 for *D’* vs. *C’*. Please see the legends of Fig. 1 for the explanations of all the abbreviations

**Fig. 3:**
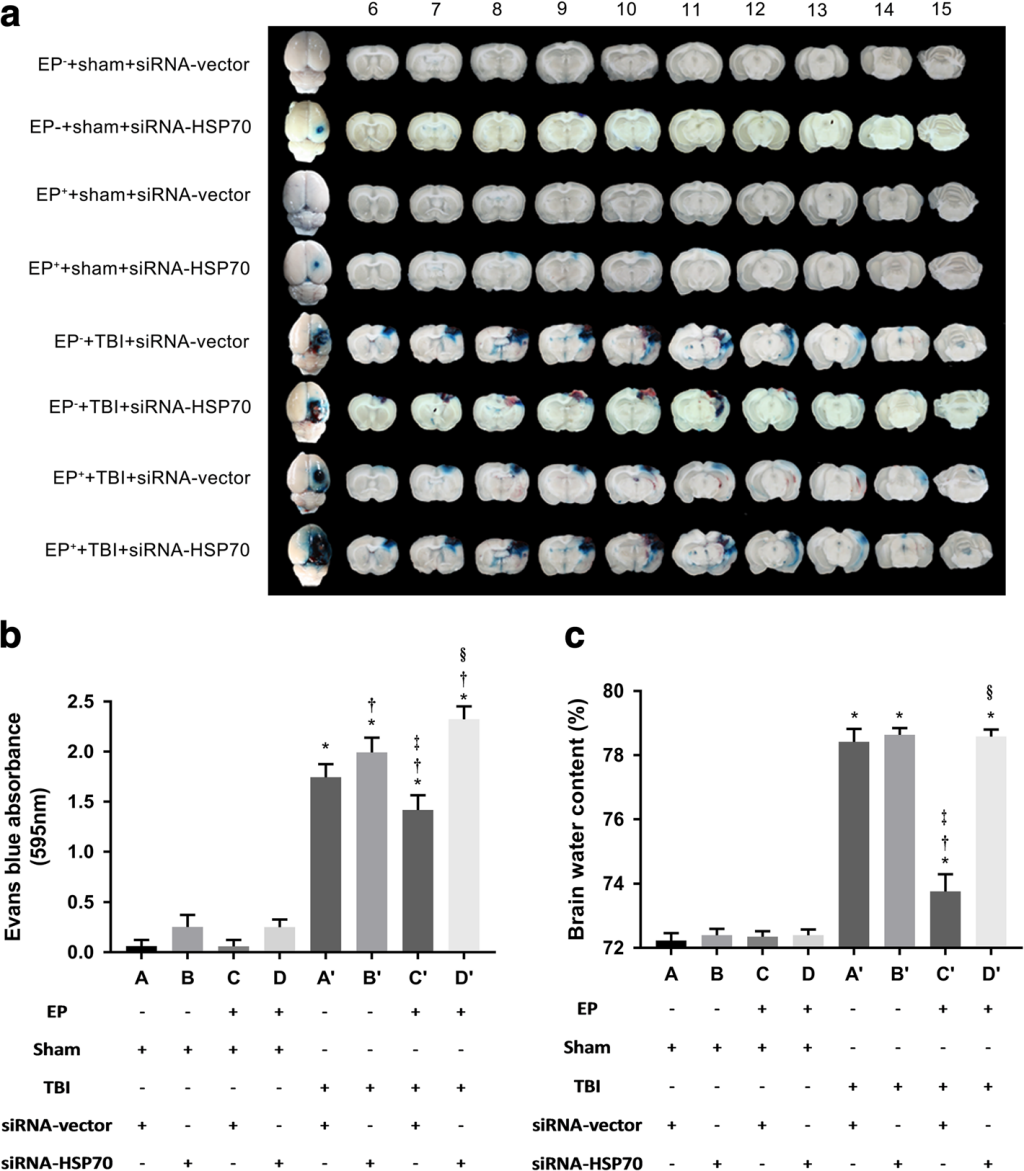
TBI-induced brain edema. Both the Evans Blue extravasation (**a**, **b**) and brain water content (**c**) presented are representatives of brain edema results. Data are presented as mean ± SD (*n* = 8 per group). *P < 0.05 for *A’* or *B’* or *C’* or *D’* vs. *A*; †*P* < 0.05 for *B’* or *C’* or *D’* vs. *A’*; ‡*P* < 0.05 for *C’* vs. *B’*; and §*P* < 0.05 for *D’* vs. *C’*. Please see the legends of Fig. 1 for the explanations of all the abbreviations

**Fig. 4:**
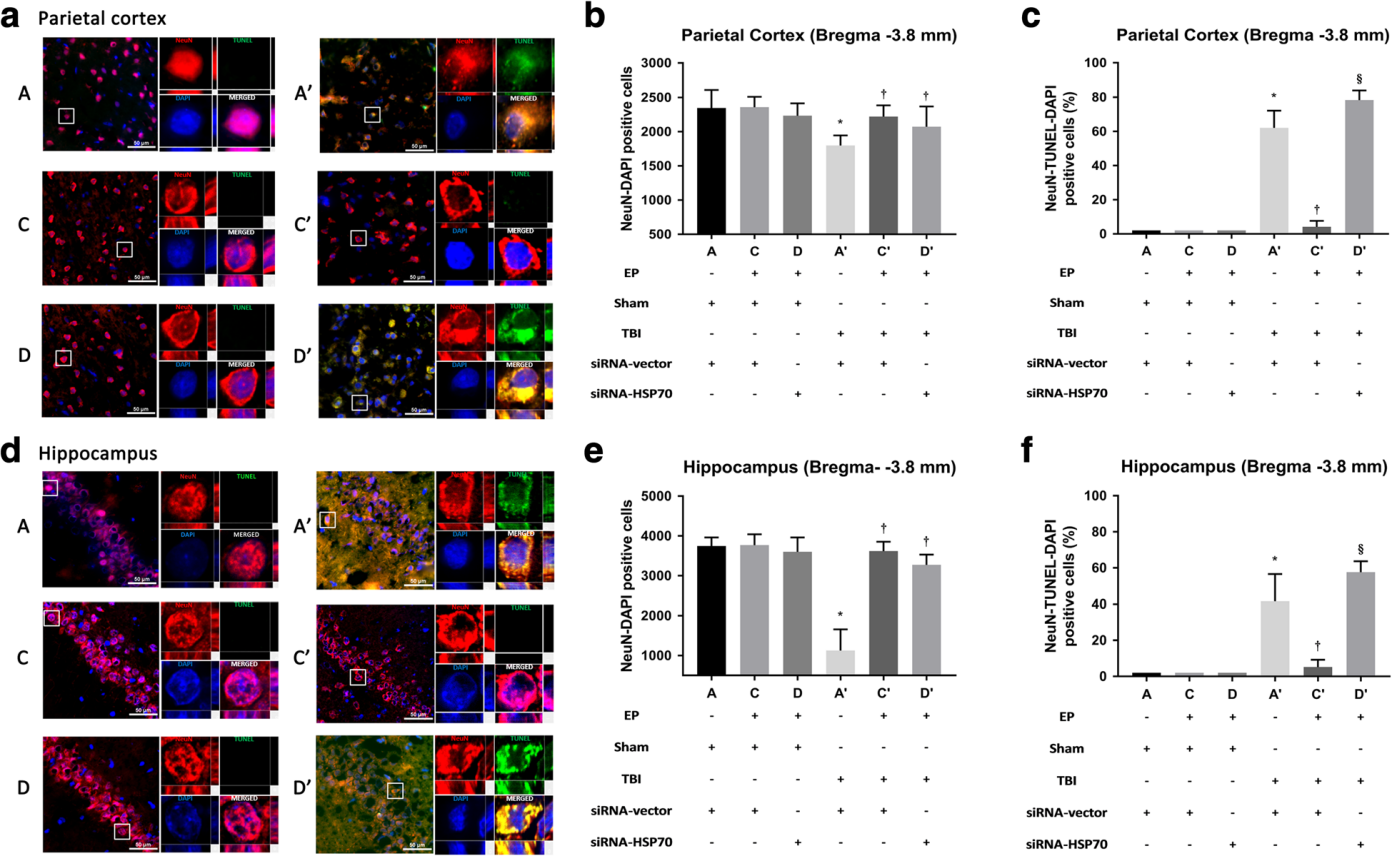
TBI-induced neuronal loss and apoptosis in both the parietal cortex and hippocampus region. TUNEL stainings are representatives of brain apoptosis (**a**, **d**). Data are presented as mean ± SD (*n* = 8 per group) (**b**, **c**, **e**, **f**). *P < 0.05 for *A’* vs. *A*; †*P* < 0.05 for *C’* or *D’* vs. *A’*; and §*P* < 0.05 for *D’* vs. *C’*. Please see the legends of Fig. 1 for the explanations of all the abbreviations

All the neurological motor deficits (Fig. 2a, b), brain contusions (Fig. 2c, d), brain edema (evidenced by increased Evans Blue extravasation and increased brain water contents) (Fig. 3a–c), and neuronal loss and apoptosis (evidenced by decreased numbers of neurons and increased numbers of apoptotic neurons respectively) (Fig. 4a–f) 3 days post-injury were all significantly attenuated in EP^+^ rats. In addition, all of the TBI-induced histopathological outcomes and neurological motor deficits in rats treated with HSP70 siRNA, but not siRNA-vector, were significantly attenuated (Figs. 2, 3, and 4). Our data provide first evidence showing the HSP-70-mediated EP protect against traumatic brain injury. Several previous investigations support the present results. For example, a single oral dose of geranylgeranylacetone upregulated brain levels of HSP70 and attenuated kainic acid-induced cognitive, affective, and sensorimotor function deficits in rats [35, 36]. In fact, a promising treatment strategy is that of preconditioning [37].

### HSP-70-mediated exercise preconditioning protects against altered blood profiles of inflammatory gene expression after brain injury

**Fig. 5:**
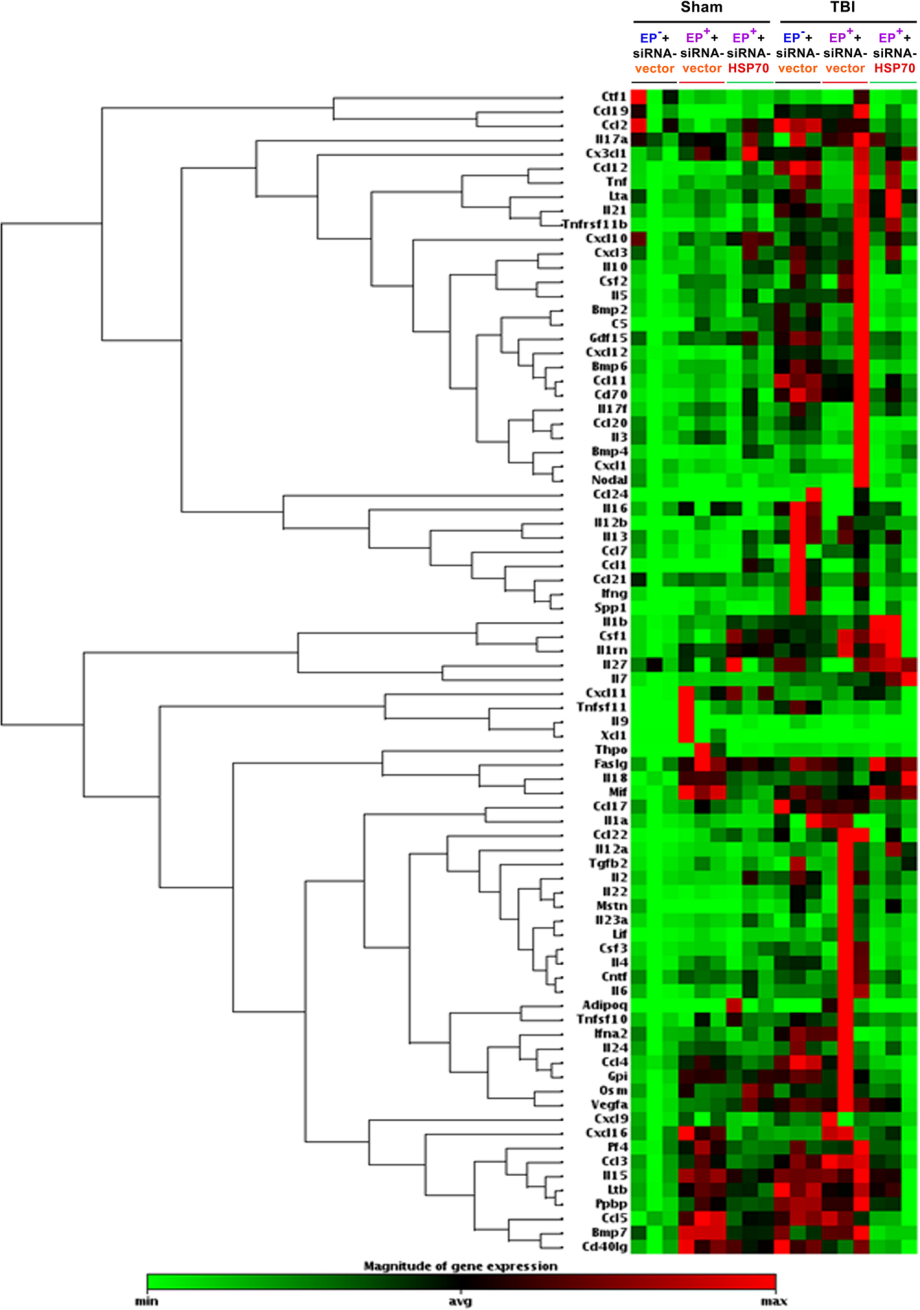
Clustering heat map of the 84 mRNA expression profiles in the peripheral blood by qPCR-based array analysis for an (EP^−^ + sham + siRNA-vector) rat, an (EP^+^ + sham + siRNA-vector) rat, an (EP^+^ + sham + siRNA-HSP70) rat, an (EP^−^ + TBI siRNA-vector) rat, an (EP^+^ + TBI + siRNA-vector) rat, and an (EP^+^ + TBI + siRNA-HSP70) rat. The samples assessed at 3 days after TBI or sham operation are listed in *columns*, and the 84 mRNAs are listed in *rows*. The relative mRNA expression is based on the color scale shown at the bottom. *Red* indicates a higher expression level; *blue* indicates lower than medium expression level abundance, and *green* indicates that mRNA expression was not detection

**Fig. 6:**
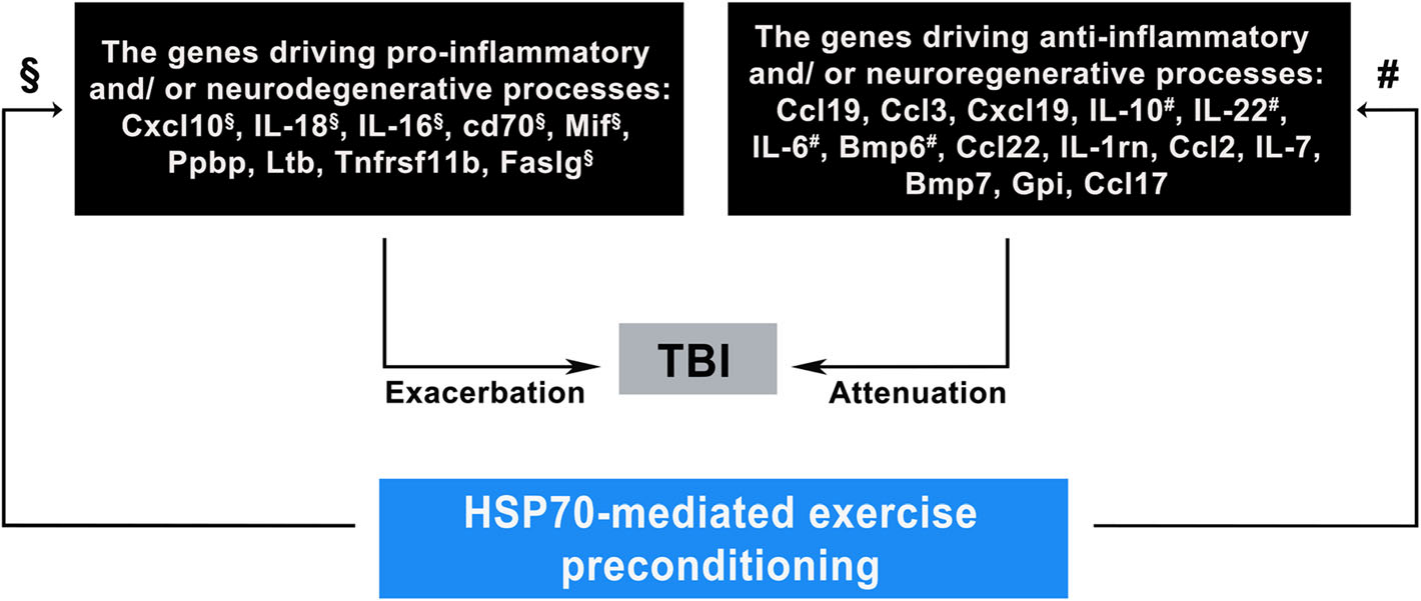
The temporal relationship driving proinflammatory and/or neurodegenerative processes vs. anti-inflammatory and/or neuroregenerative events in the blood after a TBI might be affected by HSP70-mediated EP^+^ in rats

We used a qPCR-based array to analyze 84 mRNAs expression levels in the peripheral blood at 3 days post-TBI (Fig. 5): 23 mRNAs were upregulated (Table 2). A comparison of mRNA expression profiles showed that 9 mRNAs were significantly upregulated after a TBI and were driving pro-inflammatory and neurodegenerative processes (Table 3): Cxcl10 (a promoter of detrimental effects of TBI), IL-18 (a proinflammatory cytokine), IL-16 (a proinflammatory cytokine), Cd70 (a proinflammatory cytokine), Mif (a suppressor of anti-inflammatory effects), Ppbp (a promoter of leukocyte migration), Ltb (a product of endotoxin), Tnfrsf 11b (also known as osteoprotegerin, an activator of tumor necrosis factor receptor), and Faslg (a promoter of apoptosis) (Tables 2 and 3 and Fig. 6). Moreover, 14 mRNAs driving anti-inflammatory and/or neuroregenerative events (Table 3) are also upregulated: Ccl 19, (a neuroprotective agent), Ccl 3 (an agent against microglial neurotoxicity), Cxcl 19 (an agent that induces the M1 type of macrophages or microglia into the M2 type), IL-10 (a product of M2 macrophages or microglia), IL-22 (a member of the IL-10-related cytokine), IL-6 (a product of the M2 macrophages or microglia), Bmp 6 (an inhibitor of apoptosis), Ccl 22 (a product of M2 macrophages or microglia), IL-1γn (an inhibitor of IL-1α and IL-1β), Ccl 2 (an inducer of angiogenesis), IL-7 (an anti-apoptotic agent), Bmp 7 (a neuroregenerative agent), GPi (a neurotrophic factor), and Ccl17 (also known as thymus- and activation-regulated chemokine (TARC), an anti-microbial agent). Six out of the 9 genes driving pro-inflammatory and/or neurodegenerative processes are significantly inhibited in EP^+^ + TBI rats but not in EP^−^ + TBI rats: Cxcl 10, IL-18, IL-16, Cd70, Mif, and Faslg (Table 2). In contrast, 4 out of 14 genes driving anti-inflammatory and/or neuroregenerative events after a TBI were significantly augmented in EP^+^ + TBI rats but not in EP^−^ + TBI rats: IL-10, IL-22, IL-6, and Bmp 6 (Table 2). The effects of the 6 genes driving pro-inflammatory and/or neurodegenerative processes and the 4 genes driving anti-inflammatory and/or neuroregenerative events are significantly reversed in HSP-70 gene silence rats (Tables 2 and 3 and Fig. 6). The residual 13 mRNAs upregulated after TBI were not significantly affected by EP (Table 2).

**Table 2:** The 23 genes significantly upregulated (threefold or more) in rat blood 3 days after traumatic brain injury (determined by qPCR array)

Gene symbol	Accession number	Fold increase			Gene name
		(EP^−^ + TBI + siRNA-vector)	(EP^+^ + TBI + siRNA-vector)	(EP^+^ + TBI + siRNA-HSP70)	
Chemokines					
Ccl 2	NM_031530	3.5^a^	2.50	2.08	Chemokine (c-c motif) ligand 2
Ccl 3	NM_013025	13.6^a^	7.45	8.76	Chemokine (c-c motif) ligand 3
Ccl 17	NM_057151	5.9^a^	3.3	2.8	Chemokine (c-c motif) ligand 17
Ccl 19	NM_057203	3.3^a^	−0.43	0.505	Chemokine (c-c motif) ligand 19
Ccl 22	NM_057203.1	4.208^a^	1.446^b^	3.262^c^	Chemokine (c-c motif) ligand 22
Cxcl 10	NM_139089	8.9^a^	3.95^b^	7.28^c^	Chemokine (c-x-c motif) ligand 10
Cxcl 19	NM_001113651	3.3*	−0.43	0.50	Chemokine (c-x-c motif) ligand 19
Ppbp	NM_153721	14.1^a^	9.56	10.69	Pro-platelet basic protein
Interleukins					
IL 18	NM_019165	19.3^a^	7.61^b^	14.74^c^	Interleukin-18
IL 16	NM_031512	8.6^a^	4.86^b^	31.56^c^	Interleukin-16
IL 1rn	NM_022194	2.41^a^	24.66	20.20	Interleukin 1 receptor antagonist
IL 7	NM_008760860	26.9^a^	29.26	22.83	Interleukin 7
TNF (tumor necrosis factor receptor) superfamily					
Cd70	NM_001106878	20.1^a^	2.75^b^	10.96^c^	CD70 antigen
Faslg	NM_012908	13.8^a^	8.01^b^	19.37^c^	Fasligand
Ltb	NM_212507	8.1^a^	8.4	8.8	Lymphotoxin beta
Tnfrsf 11b	NM_012870	14.3^a^	12.06	11.65	Tumor necrosis factor receptor superfamily, member 11b
Other cytokines					
Mif	NM_031051	11.9^a^	6.64^b^	10.14^c^	Macrophage migration inhibitory factor
Growth factors					
Bmp6	NM_013107	4.0^a^	15.26^b^	1.71^c^	Bone morphogenetic protein 6
Bmp7	NM_001191856	3.8^a^	4.43	3.75	Bone morphogenetic protein 7
Gpi	NM_207592	25.08^a^	27.34	25.43	Glucose-6-phosphate isomerase
Anti-inflammatory cytokines					
IL 10	NM_012854	3.70^a^	17.55^b^	2.91^c^	Interleukin 10
IL 22	NM_001191988	3.22^a^	39.33^b^	7.97^c^	Interleukin 22
IL 6	NM_012589	3.42^a^	12.49^b^	3.48^c^	Interleukin 6

Data are from 8 control (sham) and 24 TBI rats. The fold increase for each parameter for (EP^**−**^ + sham + siRNA-vector) group rats is “1”

Abbreviations: *EP* ^***−***^ *+ Sham* Sham rats that did not undergo exercise preconditioning (EP^**−**^), *EP* ^**−**^ *+ TBI* EP^**−**^ that underwent a TBI, *EP* ^*+*^ *+ TBI* EP^+^ rats that underwent a TBI, *EP* ^*+*^ *+ TBI + siRNA-vector* EP^+^ rats were treated with vector and that underwent a TBI, *EP* ^*+*^ *+ TBI + siRNA-HSP70* EP^+^ rats were treated with siRNA-HSP70 and that underwent a TBI

Only significant and annotated transcripts are included

^a^(EP^**−**^ + TBI + siRNA-vector) vs. (EP^**−**^ + sham + siRNA-vector)

^b^(EP^+^ + TBI + siRNA-vector) vs. (EP^**−**^ + TBI + siRNA-vector)

^c^(EP^+^ + TBI + siRNA-HSP70) vs. (EP^+^ + TBI + siRNA-vector)

**Table 3:** Functions of the 23 genes significantly upregulated (threefold or more) in rat blood 3 days after traumatic brain injury (determined by the qPCR array)

Gene symbol	Gene function (reference)
Chemokines	
Ccl 2	Ccl 2 signaling induces angiogenesis in the vasculature [63, 64] and protects against ischemia in the myocardium [65].
Ccl 22	Ccl 22 and IL-10 are products of M2-polarized cells [66].
Cxcl 10	Cxcl 10 expression increases in focal stroke [67] and is associated with leukocyte migration [68]. Cxcl 10 attracts T cells, which are hypothesized to be a major mechanism for the detrimental effects of TBI [69].
Ppbp (Nap.2)	NAP-2 promotes directed intravascular leukocyte migration though platelet thrombi [70].
Ccl 17	Ccl 17 possessed anti-microbial properties [71]. In cerebral ischemia, cc chemokines present an important phagocytic activity [72].
Ccl 19	In the adult central nervous system, neuroprotective and reparative proinflammatory mediator [73].
Ccl 3	CC-chemokine receptor CCR 5 activation by Ccl 3 on microglia might protect against microglial neurotoxicity [74].
Cxcl 19	In a hypoxic environment and later progression of cancer M1 macrophage produce Cxcl 19 often transition into M2 macrophages [75].
Interleukins	
IL 18	Member of the IL-1 family and pro-inflammatory cytokine [76].
IL 16	A pro-inflammatory cytokine that promotes the secretion of TNF-α, IL-1β, and IL-6 [77].
IL 1γn	Inhibits the actions of IL-1α and IL-1β [78]. In animal studies, overexpression of IL-1γn (IL-1 receptor antagonist) improves outcomes from experimental stroke [78].
IL 7	Initiates signaling cascades that induce anti-apoptotic BCL-2 family members [79].
Tumor necrosis factor receptor (TNFR) superfamily	
Cd 70	Induces the release of pro-inflammatory cytokines [80].
Faslg	Induces apoptosis [81].
Ltb	Upregulated by endotoxin in vivo [82].
Tnfrsf 11b	An indicator for poor outcome of ischemic stroke [83].
Other cytokines	
Mif	Exacerbates outcomes of experimental stroke [84].
Bmp 6	Inhibits apoptotic pathways [85].
Bmp 7	It exerts neuroprotective effects in models of stroke [86, 87].
GPi	A neurotrophic factor [88].
Anti-inflammatory cytokines	
IL-10	Provides direct trophic support to neurons [89–91]. A product of M2-polarized macrophages and microglia [66].
IL-22	A member of the IL-10-related cytokine family [92].
IL-6	A complete lack of IL-6 might be detrimental to neurogenesis in the adult brain [46, 47]. An IL-6 deficiency after TBI is associated with poor behavior performance on standard animal behavior test [45].

### HSP-70-mediated exercise preconditioning modulates a critical HSP-70/NF-κB/IL-6/synapsin I signaling system in injured brain in rats

**Fig. 7:**
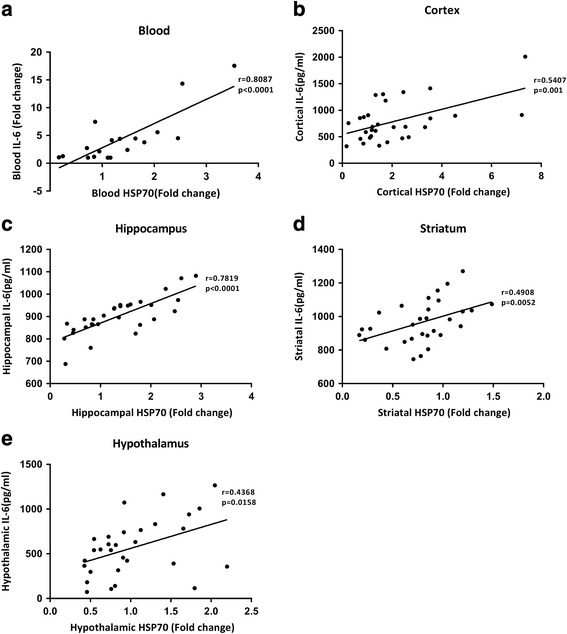
Pearson correlation between **a** the levels of IL-6 (evaluated by qPCR) and HSP70 (assessed by Western blotting) in the peripheral blood, **b** the levels of IL-6 and HSP70 (assessed by ELISA assay) in the frontal cortex, **c** the levels of IL-6 and HSP70 in the hippocampus, **d** the levels of IL-6 and HSP70 in the striatum, and **e** the levels of IL-6 and HSP70 in the hypothalamus in six groups of rats (*n* = 6 per group)

**Fig. 8:**
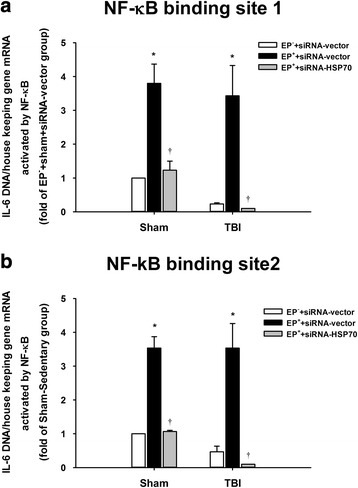
HSP70-mediated exercise preconditioning (EP^+^) induces increased expression of NF-κB in the ipsilateral frontal cortex was higher than that in HSP70-mediated EP^+^ rats. ChIP analysis was used to evaluate the extent of NF-κB binding to the DNA elements in the IL-6 promoter site 1 (**a**) and site 2 (**b**) regions using “EpiTect ChIP qPCR Assays kit” from Qiagen in (EP^−^ + sham + siRNA-vector) rats, (EP^+^ + sham + siRNA-vector) rats, (EP^+^ + sham + siRNA-HSP70) rats, (EP^−^ + TBI + siRNA-vector) rats, (EP^+^ + TBI + siRNA-vector) rats, and (EP^+^ + TBI^+^ + siRNA-HSP70) rats. The increased levels of HSP70 in front cortex caused by EP^+^ were significantly attenuated by depleting HSP70 levels with shHSP70 preconditioning. *P < 0.05 for EP^+^ + siRNA-vector vs. EP^−^ + siRNA-vector and ^†^ *P* < 0.05 for EP^+^ + siRNA-vector vs. EP^+^ + siRNA-HSP70

**Fig. 9:**
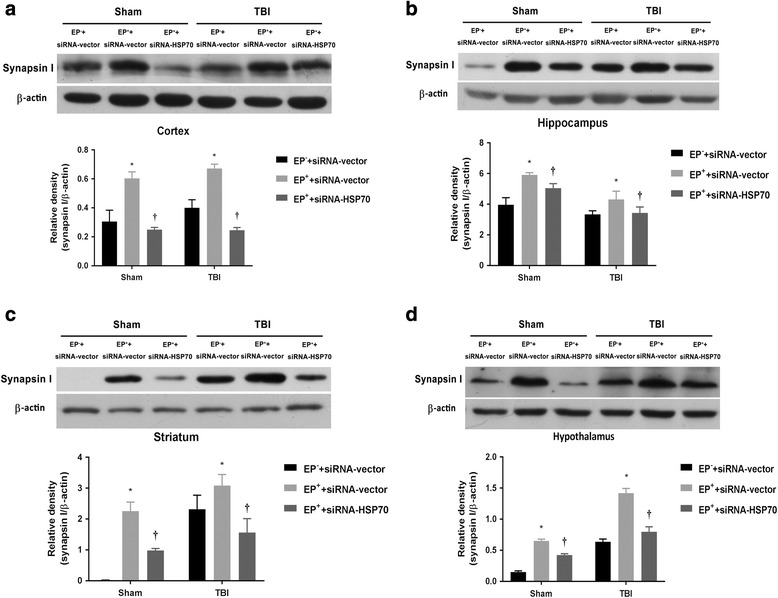
Synapsin I expression was higher in **a** the frontal cortex, **b** the hippocampus, **c** the striatum, or **d** the hypothalamus. Illustration of synapsin I protein levels in (EP^−^ + sham + siRNA-vector) rats, (EP^+^ + sham + siRNA-vector) rats, (EP^+^ + sham + siRNA-HSP70) rats, (EP^−^ + TBI + siRNA-vector) rats, (EP^+^ + TBI + siRNA-vector) rats, and (EP^+^ + TBI + siRNA-HSP70) rats 3 days after TBI determined by Western blot. The level of synapsin I is significantly (*P* < 0.05) elevated in (EP^+^ + sham + siRNA-vector) group or (EP^+^ + TBI + siRNA-vector) group over control. The increased levels of synapsin I caused by EP^+^ are significantly (*P* < 0.05) attenuated by depleting HSP70 levels with shHSP70 gene silence. Values are expressed as means ± SEM (*n* = 8 for each group) and relative to β-actin. *P < 0.05 for EP^+^ + siRNA-vector vs. EP^−^ + siRNA-vector and ^†^ *P* < 0.05 for EP^+^ + siRNA-vector vs. EP^+^ + siRNA-HSP70

Figure 7a depicts a positive Pearson correlation between the blood levels of IL-6 (measured by qPCR assay) and blood levels of HSP70 (measured by Western blotting assay). In the ipsilateral brain regions, a positive Pearson correlation also exists between cortical IL-6 (measured by ELISA) and cortical HSP70 (measured by Western blotting assay) (Fig. 7b), hippocampal IL-6 and hippocampal HSP70 (Fig. 7c), striatal IL-6 and striatal HSP70 (Fig. 7d), and hypothalamic IL-6 and hypothalamic HSP70 (Fig. 7e). The overexpression of NF-κB binding to the DNA elements in the IL-6 promoter regions in the ipsilateral frontal cortex after an EP in both TBI rats and sham-operated rats are significantly attenuated by prior depletion of cortical HSP70 with gene silence (Fig. 8a, b). Again, the overexpression of synapsin I in the ipsilateral brain regions including the frontal cortex (Fig. 9a), hippocampus (Fig. 9b), striatum (Fig. 9c), and hypothalamus (Fig. 9d) caused by EP in both TBI rats and sham-operated rats are significantly reduced by depleting cortical levels of HSP70 with gene silence (Fig. 9). According to the IPA core analysis-based network of mRNA interaction [19], a critical HSP70/NF-κB/IL-6/synapsin I signaling system in the damaged brain in rats can be confirmed by our present results.

## Discussion

In this study, using an experimental rat model, we show that EP, in addition to increasing HSP70, protects against brain contusion, edema, neuronal apoptosis, and neurological motor deficits in TBI rats. The beneficial effects of EP in treating a TBI can be attenuated by depleting cortical tissue HSP70 with gene silence. QPCR-based array analyses reveal that a TBI upregulates 23 of the 84 mRNA in the peripheral blood. A comparison of mRNA expression profiles shows that a TBI upregulates 9 mRNA (Cxcl10, IL-18, IL-16, Cd70, Mif, Ppbp, Ltb, Tnfrsf11b, Faslg), which are in the blood and drive pro-inflammatory and/or neurodegenerative processes. A TBI also upregulates the following 14 mRNAs (Ccl19, Ccl13, Cxcl19, IL-10, IL-22, IL-6, Bmp6, Ccl22, IL-1rn, Ccl2, IL-7, Bmp7, Gpi, Ccl17), which are in the blood and drive anti-inflammatory and/or neurodegenerative events. In TBI rats, EP inhibits the expression of 6 (Cxcl10, IL-18, IL-16, Cd70, Mif, and Faslg) pro-inflammatory and/or neurodegenerative genes but arguments the expression of 4 (Bmp6, IL-10, IL-22 and IL-6) anti-inflammatory and/or neuroregenerative genes. Prior depletion of cortical HSP70 with gene silence also significantly abolished the beneficial effects of EP in normalizing these altered gene expressions in the blood caused by TBI. First, our data show that a TBI upregulates the mixtures of both pro-inflammatory/neurodegenerative genes and anti-inflammatory/neuroregenerative genes. Second, we demonstrate that HSP70-mediated EP improves neurological injury by inhibiting the former genes but stimulating the later genes.

Our results confirm the current conventional wisdom that neuroinflammation is responsible for detrimental and beneficial effects that contribute to secondary TBI as well as neuro repair [7]. Monitoring inflammatory mediators using gene arrays to assay the peripheral blood of TBI patient has the potential to provide accurate information on secondary TBI and make predictions about probable outcomes. Our data suggest a possible use of cytokine profiles in the peripheral blood as biomarkers that indicate the severity of a TBI. Additionally, our results demonstrated that HSP70-mediated EP capable of redirecting the pro-inflammatory/neurodegenerative gene response toward an anti-inflammatory/neuroregenerative gene response during TBI could represent an attractive for TBI therapy.

Both in humans and in rodents, a TBI upregulates levels of both HSP70 [9] and IL-6 [16] in the central nervous system. The IPA software program has proposed that HSP70/NF-κB/IL-6/synapsin I axis be a critical inflammatory signaling system. Indeed, here, we show that a positive Pearson correlation exists between the levels of HSP70 and IL-6 in both the peripheral blood and the brain regions. A TBI upregulates the levels of IL-6 in the peripheral blood of rats. EP, in addition to enhancing expression of both HSP70 and IL-6, significantly attenuates neurological injury. Prior depletion of cortical HSP70 with gene silence significantly reverses the beneficial effects of EP in increasing cortical levels of both HSP70 and IL-6 as well as in decreasing neurological injury. The overexpression of NF-κB binding to the DNA elements in the IL-6 promoter regions in the injured brain regions after an EP in rats can also be reduced by gene silence of cortical HSP70. Prior depletion of cortical HSP70 with gene silence further attenuates overexpression of synapsin I caused by an EP in the injured brain regions of TBI rats. Our findings propose an anti-inflammatory and/or neuroprotective role for HSP70/NF-κB/IL-6/synapsin I signaling system in injured brains. EP may improve neurological injury by stimulating this signaling system.

Many previous findings support our hypothesis based on present results. For example, a moderate TBI causes blood-brain-barrier (BBB) breakdown, apoptosis and excitotoxicity, cerebral vascular pathophysiology, edema, and cerebral inflammation [38]. Physical exercise attenuates hypothalamus-pituitary-adrenal axis dysregulation, enhances neuroplasticity, and reverses the cognitive dysfunction following a TBI. Physical exercise counteracts the TBI-induced cognitive deficits by elevating the brain levels of BDNF, synapsin I, and others in the cerebral ventricles [39]. Exercise preconditioning reduces brain inflammation and protects against toxicity induced by a TBI [40]. After a TBI, IL-6, released from muscle [41], enters into the blood and then crosses the BBB [42]. Physical exercise improves TBI-induced cognitive deficits by ample release of IL-6, synapsin I, and other nerve growth factors [43]. During brain injury, both TNF-α and IL-1β exacerbates neutrophil degranulation and tissue destruction, while IL-6 inhibits both neutrophil degranulation and tissue damage caused by TNF-α and IL-1β. IL-6 can suppress the function of both TNF-α and IL-1β by increasing soluble IL-1 receptor antagonist (IL-1RA) and TNF receptor 1 (TNFR1) [44]. IL-6 knockdown mice show a compromised inflammatory, increased oxidative stress, impaired neurological activation, and a lower rate of recovery and healing following a TBI [45]. A complete lack of IL-6 might be detrimental in the adult brain [46, 47]. On the other hand, mice with overexpression of IL-6 in the brain show more rapid healing and recovery after a TBI because of extensive revascularization. Exercise differentially regulates synaptic proteins such as BDNF [48] and synapsin I [49] that play a major role in regulating learning and memory. As demonstrated in the present study, exercise preconditioning induces both inflammatory and anti-inflammatory gene responses. According to the opinion of Fehrenbach and Schneider [50], a TBI may pave the way for infectious complications such as neurological injury, whereas regular exercise may enhance immune competence for neuronal repair. Exercise preconditioning improves motor recovery after a TBI by inhibiting pro-inflammatory (IL-1β, TNF-α) cytokine accumulation and neutrophil infiltration but enhancing anti-inflammatory (IL-10) cytokines [51]. Finally, it should be stressed that although NF-κB is a major factor for neurogenesis [52], neuritogenesis [53], and synaptic plasticity [54], but this effect is evident in neurons not in glial cells. NF-κB widely plays a pro-inflammatory role in glial cells.

There are two subtypes of macrophage or microglial: M1 and M2 [2, 55–57]. The former drives pro-inflammatory/neurodegenerative processes, whereas the latter drives anti-inflammatory/neuroregenerative events [58–60]. Macrophagic and microglial responses following acute brain injury in rats are a mixture of M1 and M2 phenotypes of macrophages and microglia [56]. The damaged tissue environment after a TBI heavily favors activation of pro-inflammatory M1 microglia [12, 55, 61]. In contrast, activation of anti-inflammatory M2 microglia during traumatic brain injury mitigates damage associated with a TBI [62]. In response to lipopolysaccharide, M1 microglia can be triggered to express a variety of pro-inflammatory molecules including TNF-α, IL-1β, interferon-γ, and nitric oxide as well as cell surface markers. On the other hand, M2 polarization can be induced by IL-4. In the present results, after a TBI, the genes in the blood that drive pro-inflammatory/neurodegenerative processes and anti-inflammatory/neuroregenerative events might be derivable by activating the M1 and M2 phenotypes of macrophages and microglia, respectively, in the injured brain. Further studies are needed to ascertain whether HSP-70-mediated EP improves outcomes of TBI by transiting the phenotype of microglia and macrophage from M1 to M2.

In summary, our data depict that a positive correlation exists between the levels of HSP70 and IL-6 in both the peripheral blood and injured brain regions. The levels of IL-6 in the peripheral blood are upregulated following a rodent TBI. EP, in addition to enhancing expression of both HSP70 and IL-6, significantly attenuates neurological injury. Prior depletion of cortical HSP70 attenuates the beneficial effects of EP in reducing both overexpression of HSP70 and IL-6 and the neurological injury. Gene silence of cortical HSP70 reduced the overexpression of NF-κB binding to the DNA elements in the IL-6 promoter regions in the injured brain after an EP in rats can be reduced by gene silence of cortical HSP70. Prior depletion of cerebral HSP70 with gene silence also attenuated the overexpression of synapsin I in the brain regions of TBI rats treated with an EP.

## Conclusions

Our present data demonstrate that TBI causes neurological deficits associated with stimulating several pro-inflammatory gene profiles but inhibiting several anti-inflammatory HSP70/NF-κB/IL-6/synapsin I axis in the injured brains.

## Abbreviations

EP: Exercise preconditioning
HSP70: Heat shock protein 70
IL-6: Interleukin-6
NF-κB: Nuclear factor kappa-B
TBI: Traumatic brain injury
